# Calculating Distortions of Short DNA Duplexes with Base Pairing Between an Oxidatively Damaged Guanine and a Guanine

**DOI:** 10.3390/molecules190811030

**Published:** 2014-07-28

**Authors:** Masayo Suzuki, Katsuhito Kino, Masayuki Morikawa, Takanobu Kobayashi, Hiroshi Miyazawa

**Affiliations:** Kagawa School of Pharmaceutical Sciences, Tokushima Bunri University, 1314-1, Shido, Sanuki, Kagawa 769-2193, Japan; E-Mails: s120002@stu.bunri-u.ac.jp (M.S.); s110702@stu.bunri-u.ac.jp (M.M.); kobayashit@kph.bunri-u.ac.jp (T.K.); miyazawah@kph.bunri-u.ac.jp (H.M.)

**Keywords:** oxidatively damaged guanine, base pair, distortion of DNA duplex, destabilization energy

## Abstract

DNA is constantly being oxidized, and oxidized DNA is prone to mutation; moreover, guanine is highly sensitive to several oxidative stressors. Several oxidatively damaged forms of guanine—including 2,2,4-triamino-5(2*H*)-oxazolone (Oz), iminoallantoin (Ia), and spiroiminodihydantoin (Sp)—can be paired with guanine, and cause G:C-C:G transversions. Previous findings indicate that guanine is incorporated more efficiently opposite Oz than opposite Ia or Sp, and that these differences in efficiency cannot be explained by differences in the stabilities of G:Oz, G:Ia, and G:Sp base pairs calculated *ab* *initio*. Here, to explain previous experimental result, we used a 3-base-pair model DNA duplex to calculate the difference in the stability and the distortion of DNA containing a G:Oz, G:Ia, or G:Sp base pair. We found that the stability of the structure containing 5' and 3' base pairs adjacent to G:Oz was more stable than that containing the respective base pairs adjacent to G:Ia or G:Sp. Moreover, the distortion of the structure in the DNA model duplex that contained a G:Oz was smaller than that containing a G:Ia or G:Sp. Therefore, our discussion can explain the previous results involving translesion synthesis past an oxidatively damaged guanine.

## 1. Introduction

Mutations of genomic information are commonly caused by oxidative stress, and oxidative stressors have been implicated in aging, carcinogenesis, and many other diseases. Guanine is particularly sensitive to several oxidative stressors because it has the lowest oxidation potential among the four bases. Several different oxidative stressors can cause G:C-T:A and G:C-C:G transversions [1]. In fact, G:C-T:A and G:C-C:G transversions caused by passive smoking are detected at high frequencies in codon 12 of the *K-ras* gene [2].

**Scheme 1:**
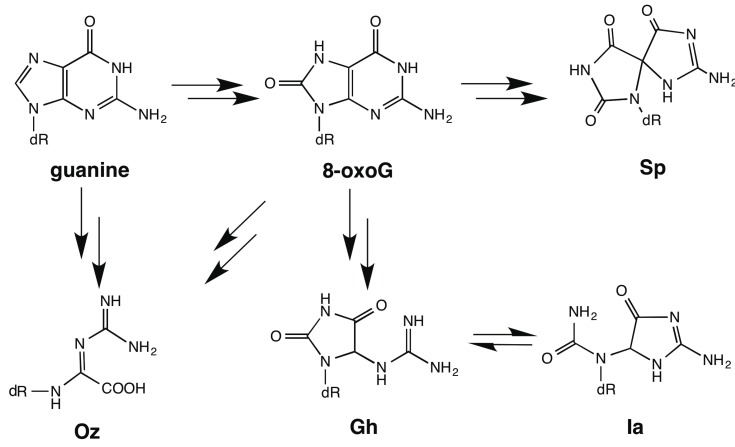
Oxidation products of guanine and 8-oxoG.

Under various oxidative conditions, guanine is oxidized and becomes 8-oxo-7,8-dihydroguanine (8-oxoG) (Scheme 1). Because 8-oxoG can be paired with adenine, but not guanine, 8-oxoG: A base pairs cause only G:C-T:A transversions [3]. Therefore, G:C-C:G transversions are presumably caused by oxidatively damaged guanine. Guanine and 8-oxoG can each be oxidized to 2,2,4-triamino-5(2*H*)-oxazolone (Oz) (Scheme 1) [4]. During DNA replication, DNA polymerase (Pol) α, β, γ, ε, η, I, or IV commonly incorporates guanine opposite Oz [5,6,7].

**Figure 1:**
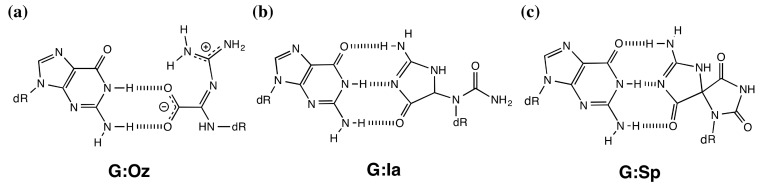
The proposed hydrogen bonding of G:Oz, G:Ia, and G:Sp base pairs.

Guanidinohydantoin (Gh) and spiroiminodihydantoin (Sp) can be formed via oxidation of 8-oxoG (Scheme 1). Gh is a principal product of 8-oxoG oxidation under acidic conditions, and Sp is a major product under basic conditions [8,9,10,11]. Gh isomerizes to iminoallantoin (Ia) [12], but whether the predominant isomer is Gh or Ia during DNA polymerization is unknown [12]. Based on our previously published calculations of the stability of G:Ia and G:Gh base pairs, Gh is predicted to tautamerize to Ia when guanine is inserted opposite Gh/Ia [13]. Hence, in this study, we consider only Ia. Incorporation of guanine opposite Oz is more efficient than that opposite Gh/Ia or Sp, and translesion synthesis past Oz is also more efficient than that past Gh/Ia or Sp [5,7,14]. However, depending on the number of hydrogen bonds, a G:Oz base pair was less stable than either a G:Ia or the G:Sp base pair in our calculated results (Figure 1) [13]. 

Generally, since replicative DNA polymerases are highly susceptible to geometric distortions in DNA, they have difficulty synthesizing past distorting DNA damage [15]. The previous study showed that the geometry of DNA containing nonplanar DNA damage readily destabilizes and distorts relative to DNA that includes natural Watson-Crick base pairs [16]. Therefore, to resolve the discrepancy between experimental results and calculated results, we focused this study on differences among the distortion of DNA containing these lesions. As depicted in Figure 1, the G:Oz base pair has no *sp^3^* carbon and is planar, while G:Ia and G:Sp base pair have a *sp^3^* carbon and are nonplanar. Ia and Sp break the planarity of the adjacent base pairs due to a *sp^3^* carbon, and as a result, DNA containing Ia or Sp is easily distorted compared to that containing Oz.

In this study, we tried to explain the experimental results by calculating the differences in the stabilities and distortions of DNA containing a G:Oz, G:Ia, or G:Sp base pair relative to DNA containing a G:C. We use 3-base-pair model DNA duplexes that each contained a central G:Oz, G:Ia, or G:Sp base pair as a way to calculate the differences in stability and distortion.

## 2. Results and Discussion

### 2.1. The Minimized Structures of DNA Duplex Containing an X:G Base Pair 

We previously calculated stabilization energies of oxidatively guanine damage (Oz, Ia or Sp) paired with guanine [13]. Since Ia and Sp have a *sp^3^* carbon, they have *S* and *R* stereoisomers. Our calculated results showed that the stabilization energies of the base pairs, “G:X (where X = Oz, *S*-Ia, *R*-Ia, *S*-Sp, or *R*-Sp)”, are ordered as follows: G:*S*-Ia > G:*R*-Ia >> G:*R*-Sp > G:*S*-Sp >> G:Oz [13]. Here, we investigated differences in stabilities of duplexes containing one G:X base pair in the active site of DNA polymerase; each Pol β-DNA complex containing a G:X base pair was built by modification of the Pol β ternary complex (PDB entry 1BPY) [17]. We used the structure of G:X base pair formed the most stable base pair with guanine based on our previous report [13].

**Figure 2:**
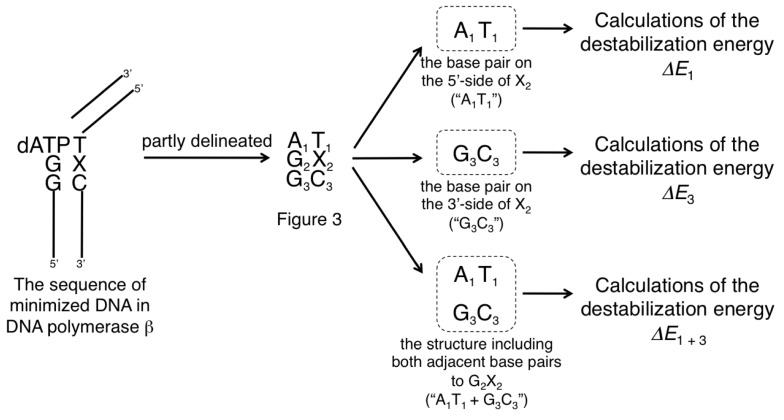
An overview of calculating the destabilization energies of DNA duplexes. Each Pol β-DNA complex containing a G:X (where X = C, Oz, *S*-Ia, *R*-Ia, *S*-Sp or *R*-Sp) base pair was minimized. G:X and each base pair adjacent to G:X is delineated in Figure 3. A:T base pair on the 5'-side of X was designated “A_1_T_1_”, G:X base pair was designated “G_2_X_2_”, and G:C base pair on the 3'-side of X was designated “G_3_C_3_”. The destabilization energies of “A_1_T_1_” (*ΔE_1_*), “G_3_C_3_” (*ΔE_3_*), and “A_1_T_1_ + G_3_C_3_” (*ΔE_1 + 3_*) were calculated *ab initio* as the parts common to each model duplex; each G_2_X_2 _base pair was excluded from the calculations.

**Figure 3:**
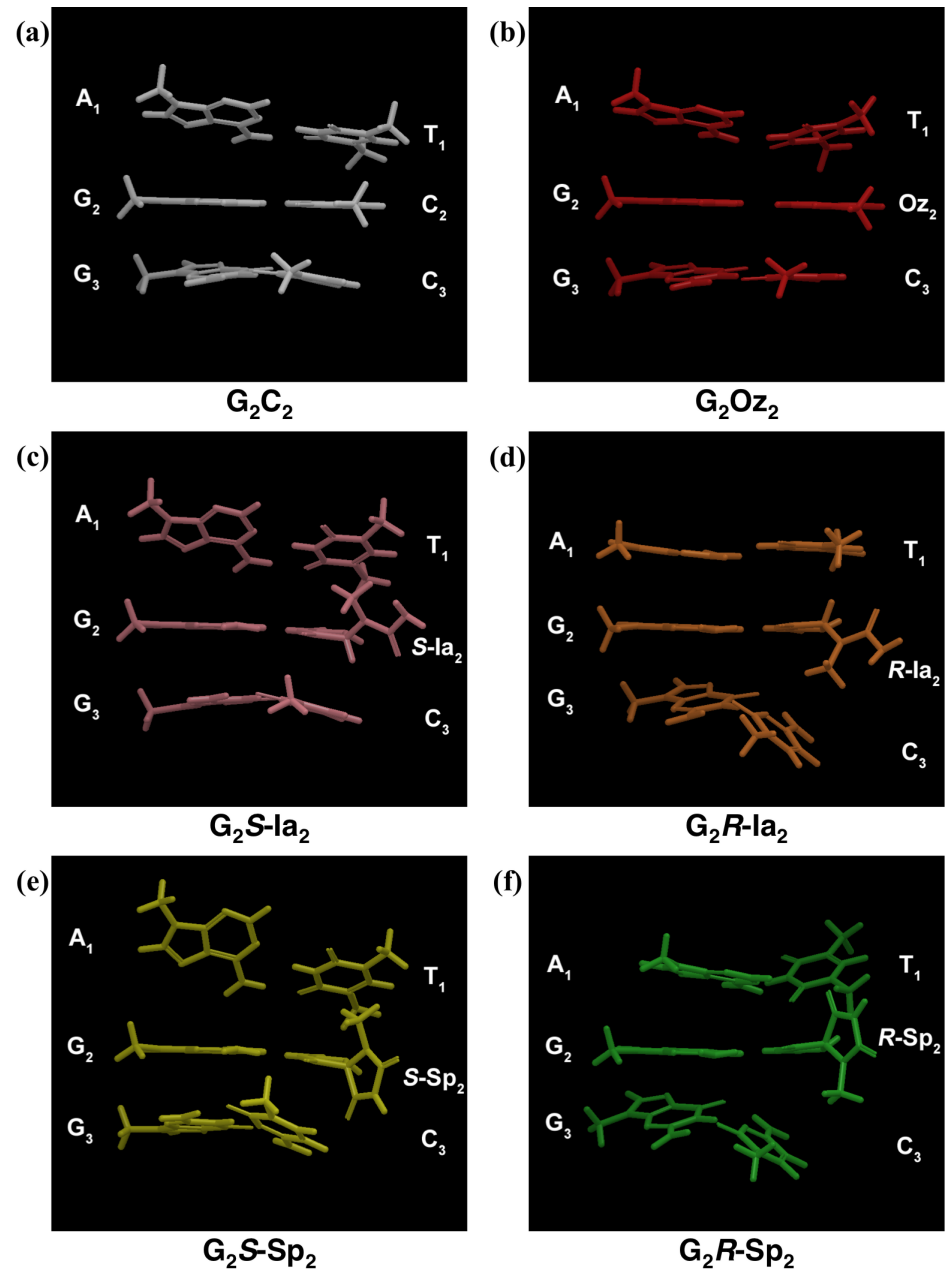
Minimized geometries of “A_1_T_1_, G_2_X_2_, G_3_C_3_” containing X_2_ = (**a**) C, (**b**) Oz, (**c**) *S-*Ia, (**d**) *R*-Ia, (**e**) *S-*Sp, or (**f**) *R-*Sp as viewed from the minor groove.

Quantum mechanical (QM) calculations are more quantitative than molecular mechanics (MM) calculations. In this study, we used QM calculations only to optimize the most important part of each G:X base pair. However, the Pol β-DNA complex model had too many atoms to use QM calculations for minimizing the model in an experimentally acceptable amount of time. Therefore, we used MM calculations, which are quantitatively inferior to QM calculations, to minimize the Pol β-DNA model. Only the most important part of each G:X base pair was optimized by QM calculation, and the geometries of Pol β-DNA complex model were minimized via MM calculations. We focused on the G:X base pair and each of two adjacent base pairs, one 5' and one 3' to the central G:X base pair, in each minimized structure (Figure 2 and Figure 3). In Figure 2, the central G:X base pair was designated “G_2_X_2_”; the 5' A:T base pair was designated “A_1_T_1_”, and the 3' G:C base pair was designated “G_3_C_3_”. Minimized geometries of A_1_T_1_, G_2_X_2_ and G_3_C_3_ base pairs are depicted in Figure 3. We wanted to compare the impact of each X_2_ on the calculated energies of the duplexes. However, the X_2 _variants differ from one another in the number of atoms; therefore, the energies of the structures containing different X_2_ could not be compared directly. Therefore, we focused on the energies of the elements common to each duplex (*i.e.*, A_1_T_1 _and G_3_C_3_) to compare among the energies of the duplexes. By calculating the energies of the common elements and excluding each “G_2_X_2_” base pair within each structure, we assessed the destabilization caused by the oxidatively damaged guanine.

### 2.2. The Destabilization Energies of the Base Pairs on 5'- and 3'-Side of X

In this section, in order to evaluate the destabilization of a DNA duplex caused by particular forms of oxidatively damaged guanine, we calculated the destabilization energies of elements common to each structure and excluded each “G_2_X_2_” base pair.

#### 2.2.1. The Destabilization Energies of the Base Pairs on 5'- Side of X

We calculated the destabilization energies of each “A_1_T_1_” (*ΔE_1_*) on the 5'-side of each X_2_. The calculated *ΔE_1_* value associated with duplexes that contained Oz, *S-*Ia, *R-*Ia, *S-*Sp, or *R-*Sp were defined by Equation (1) (Experimental Section) and were calculated *ab initio* (Table 1). Values of *ΔE_1_ in vacuo* or in water were calculated, and were presented as *ΔE_1_*^DFT^ and *ΔE_1_*^SCRF^, respectively. The values of *ΔE_1_*^DFT^ were ordered as follows with respect to each X_2_: *R*-Sp > *S*-Sp > *S*-Ia > Oz > *R*-Ia. The order of *ΔE_1_*^SCRF^ values in water was the same as the order of *ΔE_1_*^DFT^ values *in vacuo*.

**Table 1:** Destabilization energies (kcal/mol) of “A_1_T_1_” (*ΔE_1_*), “G_3_C_3_” (*ΔE_3_*), and “A_1_T_1_ + G_3_C_3_” (*ΔE_1 + 3_*), each value was calculated with minimized geometries.

		A_1_T_1_			G_3_C_3_			A_1_T_1_ + G_3_C_3_	
X *^a^*		*ΔE_1_*^DFT^	*ΔE_1_*^SCRF^		*ΔE_3_*^DFT^	*ΔE_3_*^SCRF^		*ΔE_1 + 3_*^DFT^	*ΔE_1 + 3_*^SCRF^
Oz		1.3	1.1		−0.1	0.6		1.1	1.0
*S-*Ia		1.4	1.3		2.6	1.3		4.1	4.5
*R-*Ia		0.5	0.6		4.0	3.9		4.8	4.6
*S-*Sp		2.1	2.1		2.7	2.8		4.8	5.3
*R-*Sp		12.6	12.4		5.3	4.5		18.3	18.3

*^a^* X = the damage contained in the minimized structure.

Using kinetic parameters, Kornyushyna *et al.,* demonstrated that incorporation of guanine opposite Gh/Ia is more efficient than that opposite Sp [14]. Our calculated data indicated that “A_1_T_1_” on the 5'-side of Ia was more stable than that of Sp regardless of the stereoisomer, and our data corresponded with these published results. However, our data indicated that “A_1_T_1_” on 5'-side of Oz was less stable than “A_1_T_1_” 5' to *R-*Ia, and this finding could not account for the previous experimental finding that translesion synthesis past Oz is more efficient than that past Ia [5]. Therefore, we calculated the destabilization energies for each G_3_C_3_ base pair on the 3'-side of each X_2_.

#### 2.2.2. The Destabilization Energies of the Base Pairs on the 3'-Side of X

The destabilization energies (*in vacuo*; *ΔE_3_*^DFT^ and in water; *ΔE_3_*^SCRF^) of “G_3_C_3_” were defined by Equation (2) (Experimental Section), and were calculated individually (Table 1). The values of *ΔE_3_*^DFT^ and *ΔE_3_*^SCRF^ were ordered as follows with regard to each X_2_: *R*-Sp > *R*-Ia > *S*-Sp > *S*-Ia > Oz. Surprisingly, the calculated values for *ΔE_3_*^DFT^ and *ΔE_3_*^SCRF^ of Oz were the lowest among the values for any X_2_, and “G_3_C_3_” on 3'-side of Oz was the most stable. 

The stabilities of the “G_3_C_3_” base pairs on the 3'-side of X_2_ were consistent with previous experimental results indicating that translesion synthesis past Oz is more efficient than that past Ia or Sp [5,14]. The “G_3_C_3_” base pairs on 3'-side of *S*-Sp was more stable than that of *R*-Ia; consequently, it was not possible to fully explain the previous experimental results based on the stabilities of G_3_C_3_ base pairs on the 3'-side of X_2_. Consequently, we then considered the stability of the larger structure by examining the energies of the structure containing both base pairs (5' and 3') adjacent to each X_2_.

#### 2.2.3. The Destabilization Energy of Each Model Duplex was Calculated by Including both Adjacent Base Pairs and Excluding the Central “G_2_X_2_” Base Pair

The destabilization energy of A_1_T_1_ + G_3_C_3_ (*in vacuo*;* ΔE_1 + 3_*^DFT^ and in water; *ΔE_1 + 3_*^SCRF^) was calculated for each structure, including both adjacent base pairs and excluding the central “G_2_X_2_” base pair. These values were calculated with Equation (3) (Experimental Section) (Table 1). The lowest *ΔE_1 + 3_*^DFT^ and *ΔE_1 + 3_*^SCRF^ values among all values calculated for any X_2_ were the values for Oz. Values of *ΔE_1 + 3_*^DFT^ and *ΔE_1 + 3_*^SCRF^ for Ia were lower than those for Sp, and *S* configurations were more stable than *R* configurations for both Ia and Sp. Taken together, values of *ΔE_1 + 3_*^DFT^ for the guanine oxidation products were ordered as follows for the X_2_ variants: *R*-Sp >* S*-Sp~*R*-Ia > *S*-Ia > Oz, and values of *ΔE_1 + 3_*^SCRF^ were ordered as follows: *R*-Sp >* S*-Sp > *R*-Ia > *S*-Ia > Oz. 

“A_1_T_1_ + G_3_C_3_” with Oz was more stable than that with any other X_2_; this finding indicated that a DNA structure containing G_2_Oz_2_ was more similar to DNA containing G_2_C_2_ than was that with any other G_2_:X_2 _base pair. As mentioned in the introduction; the geometry of DNA that includes nonplanar DNA damage is easy to destabilize and distort relative to that containing natural Watson-Crick base pairs [16]; and DNA polymerases have difficulty synthesizing through distorted DNA [15]. Therefore; our calculated values could account for the previous experimental findings that translesion synthesis across Oz is more efficient than that across Ia or Sp [5,14].

Notably, the calculated *ΔE_1__ + __3_*^DFT^ values for *R*-Ia and *S*-Sp were not consistent with previous results [14]. Actual experiments involving DNA polymerization have been performed in aqueous solution [5,14]; therefore, the stability of DNA duplex during DNA polymerization is attributed to the energy in water (*ΔE_1 + 3_*^SCRF^).* ΔE_1 + 3_*^SCRF^ for *R*-Ia was lower than *ΔE_1 + 3_*^SCRF^ for *S*-Sp, which can account for the previous result [14]. Furthermore, the calculated stabilities of “A_1_T_1_ + G_3_C_3_” with* S*-Sp or *R*-Sp could explain why bypass of *S*-Sp is more efficient than bypass of *R*-Sp *in vivo* [18].

In conclusion, although calculating either energies of “A_1_T_1_” on the 5'-side of X_2_ or energies of “G_3_C_3_” on the 3'-side of X_2_ was not sufficient, calculating energies of the structure while including both base pairs, “A_1_T_1_ + G_3_C_3_”, adjacent to “G_2_X_2_” was sufficient to evaluate the stability of the DNA duplex containing an G:Oz, G:Ia, or G:Sp base pair. In the next section, we used parameterized distortion of DNA to examine the effects of the non-planarity of Oz, *S-*Ia, *R-*Ia *S-*Sp, or *R*-Sp on A_1_T_1_ base pairs and G_3_C_3_ base pairs in DNA duplexes that each contain one central G:X base pair. 

### 2.3. The Degree of Distortion from DNA Duplex Containing a G:C Base Pair

Based on the images in Figure 3, we received the visual impression that there were differences in the degree of distortion among the DNA duplexes that contained C, Oz, *S-*Ia, *R-*Ia *S-*Sp, or *R*-Sp. Because abstract visual impressions were insufficient for detailed comparisons among the structures, it was necessary to parameterize actual values for those visual impressions. In this section, we examined differences in the degree of distortion caused by each G:X base pair to parameterize the distortion of each DNA duplex.

#### 2.3.1. The Degree of Distortion at the 5'-Side of X

**Figure 4:**
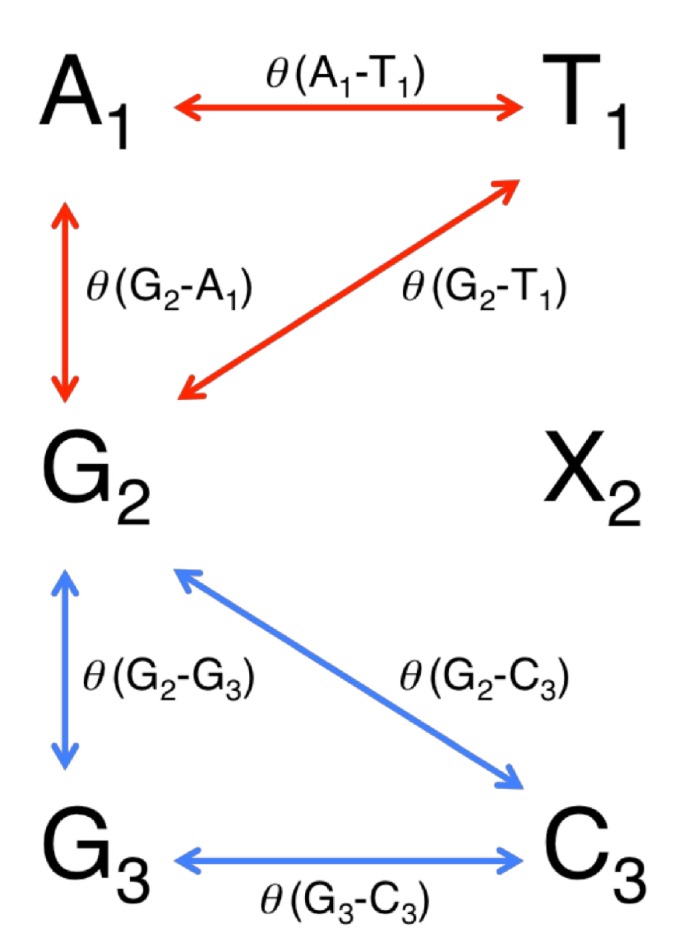
Calculated dihedral angle *θ* (G_2_–A_1_), *θ* (G_2_–T_1_) and *θ* (A_1_–T_1_) showed red arrows, and the calculated dihedral angle *θ* (G_2_–G_3_), *θ* (G_2_–C_3_) and *θ* (G_3_–C_3_) showed blue arrows.

In order to determine the degree of distortion at the 5'-side of each G_2_X_2 _base pair, we calculated the dihedral angles (*θ*) between G_2_ and A_1_ (*θ* (G_2_–A_1_)) or G_2_ and T_1_ (*θ* (G_2_–T_1_)) or A_1_ and T_1_ (*θ* (A_1_–T_1_)) (red arrows in Figure 4). Values for *θ* (G_2_–A_1_), *θ* (G_2_–T_1_), and *θ* (A_1_–T_1_) were each calculated individually via Equations (5)–(7), respectively (Experimental Section). The degree of distortion (*δ_1_*) on the 5'-side of G_2_X_2_ was defined as the sum of *θ* (G_2_–A_1_), *θ* (G_2_–T_1_), and* θ* (A_1_–T_1_) (Equation (11) in Experimental Section). In Table 2, the values of *δ_1_* were ordered as follows with regard to X_2_ variants: *S*-Sp* > R*-Sp >* S*-Ia > Oz > C > *R*-Ia. 

**Table 2:** Dihedral angles *θ* (G_2_–A_1_), *θ* (G_2_–T_1_), and *θ* (A_1_–T_1_) (red arrows in Figure 4), and the degree of distortion *δ_1_*.

X *^a^*	*θ* (G_2_–A_1_)	*θ* (G_2_–T_1_)	*θ* (A_1_–T_1_)	*δ_1_*
C	25.2°	12.5°	13.3°	51.0°
Oz	19.4°	18.2°	18.0°	55.6°
*S-*Ia	38.3°	28.2°	11.3°	77.8°
*R-*Ia	3.3°	4.0°	4.0°	11.3°
*S-*Sp	56.1°	29.9°	26.4°	112.4°
*R-*Sp	10.9°	32.4°	43.2°	86.5°

*^a^* X = the damage contained in the minimized structure.

Based on the values of *δ_1_*, the distortion of “A_1_T_1_” base pair adjacent to *R-*Ia is the smallest, and this structure was similar to the structure of the “A_1_T_1_” derived from DNA duplex containing a G_2_C_2_ (Table 1, Figure 3a,d). Consequently, the calculated *ΔE_1_* for *R-*Ia was the lowest for all X_2 _variant. Furthermore, the order of calculated *ΔE_1 _*values with respect to X_2_ variants—*S*-Ia > Oz > *R*-Ia—coincided with the order of *δ_1_*. Thus, since a large *δ_1_* indicates a prominent distortion of A_1_T_1_, we believe that the destabilization of A_1_T_1_ base pair could result from by the distortions caused by the X_2_ variants. However, *ΔE_1_* values with *R*-Sp or* S*-Sp could not be explained by *δ_1_* caused by *R*-Sp or *S*-Sp. Furthermore, *δ_1_* values for* S* configurations were larger than those for *R* configurations both for Ia and for Sp. The C1' methyl of *S*-Ia or* S*-Sp was oriented in the direction of the “A_1_T_1_” base pair (Figure 3c,e); therefore, the effects of the *S* configurations on 5'-side of X_2_ were larger than those of the *R* configurations. In summary, *δ_1_* values could not explain previous experimental results, nor could they completely account for *ΔE_1 _*values.

#### 2.3.2. The Degree of Distortion at the 3'-Side of X 

As with 5'-side of X_2_ (in Section 2.3.1), we calculated the dihedral angle *θ* between G_2_ and G_3_ (*θ* (G_2_–G_3_)), or G_2_ and C_3_ (*θ* (G_2_–C_3_)), or G_3_ and C_3_ (*θ* (G_3_–C_3_)) (blue arrows in Figure 4). Values for *θ* (G_2_–G_3_), *θ* (G_2_–C_3_), and *θ* (G_3_–C_3_) were each calculated individually via Equations (8)–(10), respectively (Experimental Section). The degree of distortion (*δ_3_*) at the 3'-side of X_2_ was defined as the sum of *θ* (G_2_–G_3_), *θ* (G_2_–C_3_), and *θ* (G_3_–C_3_) (Equation (12) in Experimental Section). All numeric values are listed in Table 3. For the X_2_ residues, the order of values for *δ_3_* was as follows: *R*-Ia > *R*-Sp > *S*-Sp > *S*-Ia > C > Oz.

**Table 3:** Dihedral angles *θ* (G_2_–G_3_), *θ* (G_2_–C_3_), and *θ* (G_3_–C_3_) (blue arrows in Figure 4), and the degree of distortion *δ_3_*.

X *^a^*	*θ* (G_2_–G_3_)	*θ* (G_2_–C_3_)	*θ* (G_3_–C_3_)	*δ_3_*
C	9.5°	8.0°	17.2°	34.6°
Oz	11.1°	3.1°	11.9°	26.1°
*S-*Ia	5.4°	13.9°	19.2°	38.5°
*R-*Ia	17.9°	42.6°	49.2°	109.7°
*S-*Sp	5.5°	25.7°	23.2°	54.4°
*R-*Sp	22.9°	36.2°	43.3°	102.5°

*^a^* X = the damage contained in the minimized structure.

When comparing among the X_2_ variants, the “G_3_C_3_” adjacent to Oz was the least distorted based on the values of *δ_3_*, and the most similar in structure to the “G_3_C_3 _in a DNA duplex containing a G_2_C_2_ (Figure 3a,b). Additionally, the calculated *ΔE_3_* with Oz was lowest, and the order of X_2_ variants with regard to calculated *ΔE_3_* values—*S*-Sp > *S*-Ia > Oz (Table 1)—was consistent with the order of *δ_3 _*values. As was the case with *δ_1_* (showed in Section 2.3.1), the destabilization of the G_3_C_3_ base pair could be accounted for by the distortion caused by the G_2_X_2_ base pair. In contrast to *δ_1 _*values*,* *δ_3_* values associated with* R* configurations were larger than those associated with *S* configurations for both Ia and Sp; moreover, the C1' methyl of *R*-Ia or* R*-Sp was oriented in the direction of the “G_3_C_3_” base pair (Figure 3d,f). Consequently, the C_3_ base of *R*-Ia or* R*-Sp was particularly distorted by the C1' methyl of *R*-Ia or* R*-Sp, respectively. Taken together, *δ_3_* values could not account for *ΔE_3_* values or previous experimental results, as was the case for *δ_1_* values.

#### 2.3.3. The Total Degree of Distortion at the 5'-Side and 3'-Side of X

The total degree of distortion (“*δ_1_ +* *δ_3_*”) at the 5'- and 3'-side of X_2_ was defined as the sum of *δ_1_* and *δ_3_* (Table 4). The “*δ_1_ +* *δ_3_*” values were ordered as follows with regard to X_2_ variants: *R*-Sp* > S*-Sp > *R*-Ia > *S*-Ia > C > Oz. This order was consistent with the order of the calculated energies (*ΔE_1_*_* + 3*_). Although the value of “*δ_1_ +* *δ_3_*” for Oz was smaller than that for C, the structure of “A_1_T_1_ + G_3_C_3_” adjacent to “G_2_Oz_2_” was less stable than that to“G_2_C_2_” according to *ΔE_1_*_* + 3*_: The stabilities of the structure of “A_1_T_1_ + G_3_C_3_” were only partially explained by the degree of distortion.

**Table 4:** Total degree of distortion (“*δ_1_* *+* *δ_3_*”).

*X ^a^*	*δ_1_ +* *δ_3_*
C	85.6°
Oz	81.7°
*S-*Ia	116.3°
*R-*Ia	121.1°
*S-*Sp	166.8°
*R-*Sp	189.0°

*^a^* X = the damage contained in the minimized structure.

#### 2.3.4. The Difference Between the Degree of Distortion for Oxidatively Damaged Guanine and that for C

We tried to eliminate the discordance between the distortion and the stability in “G_2_Oz_2_” and “G_2_C_2_”. Instead of a simple comparison among the values of the degree of distortion for any X_2_, we calculated the difference between the value of *δ_1_* *+* *δ_3_* for C and that for Oz, *S*-Ia,* R*-Ia, *S*-Sp or* R*-Sp as well as the destabilization energy. We found that the values of *δ_1_* *+* *δ_3_* for* S*-Ia or* R*-Ia differed from that for C by 30.7° or 35.4°, respectively; in contrast, there was only slight difference of 4.0° between that for Oz and that for C. The distortion of the structure of “A_1_T_1_ + G_3_C_3_” adjacent to “G_2_Oz_2_” was smaller than that adjacent to “G_2_*S*-Ia_2_” or “G_2_*R*-Ia_2_”, which seemed consistent with visual impressions.Additionally, the values of *δ_1_* *+* *δ_3_* for *S*-Sp or* R*-Sp differed from that of C by 81.1° or 103.3°, respectively. These values indicated that the structures of “A_1_T_1_ + G_3_C_3_” adjacent to “G_2_*S*-Sp_2_” or “G_2_*R*-Sp_2_” were the most distorted from that adjacent to “G_2_C_2_”; these findings seemed consistent with the visual impression of the images in Figure 3. These discussions accounted for the differences among the calculated energies (*ΔE_1 + 3_*) of the structure of “A_1_T_1_ + G_3_C_3_” adjacent to “G_2_X_2_”.

In addition to the values of *δ_1_* *+* *δ_3_*, the values of *δ_1_* or *δ_3_* for Oz, *S*-Ia,* R*-Ia, *S*-Sp, or* R*-Sp were compared with that for C. The distortions of each X_2_-containing structure compared to the value of *δ_1_* for C was ordered as follows with regard to X_2_ variants: *S*-Sp > *R*-Ia > *R*-Sp* > S*-Ia > Oz: That of *δ_3_* was ordered as follows: *R*-Ia > *R*-Sp >* S*-Sp > Oz *> S*-Ia. Therefore, the distortion for each X_2 _compared the value of *δ_1_* or* δ_3_* for C was not consistent with the visual impression, unlike the values of *δ_1_* *+* *δ_3_*, which were consistent with the visual impressions. 

In summary, the previous experimental results were only partially explained by either *δ_1_* or *δ_3_* (Section 2.3.1 and Sections 2.3.2); however, “*δ_1 _+* *δ_3_*” compensated for the weaknesses of each single measure by summing *δ_1_* and *δ_3_*. Consequently, “*δ_1_ +* *δ_3_*” was able to account for the previous experimental results and *ΔE_1 + 3_*. According to our data, the structure of “A_1_T_1_ + G_3_C_3_” adjacent to “G_2_X_2_” was distorted by the effects of the non-planarity of each lesion; in other words, the instability of a DNA duplex containing oxidatively damaged guanine was determined by the non-planarity of each oxidatively damaged guanine. Thus, it seemed reasonable that the projected distortion of a DNA duplex containing a G:Oz base pair would be the smallest.

## 3. Experimental

### 3.1. Molecular Modeling 

In this study, we constructed models of Pol β-DNA complexes containing a G:X (where X = C, Oz, *S*-Ia, *R*-Ia, *S*-Sp, or *R*-Sp) base pair using the structure (PDBID:1BPY) [17]. The details of method used to construct these models are presented below. In each model, the incoming nucleotide (dCTP) was replaced with dATP, and the template G for the incoming nucleotide was replaced with T. Each complex containing Pol β and a G:X base pair was built by replacing the G:C base pair at the 3' terminus of the template-primer with G:X base pair optimized previously [13]. The amino acid residues and other DNA base sequences were unchanged. The geometry of each atom was minimized at the OPLS2005/water level using Macromodel 9.0 (Schrödinger LLC, New York) with the fixed G:X base pair.

### 3.2. ab Initio Calculations 

For each minimized structure, all atoms were removed from the Pol β-DNA model except for (1) constituents of the bases of each G:X base pair, (2) constituents of the bases of each base pair adjacent to the G:X base pair, (3) the 2-deoxyribose C1' carbon, and (4) the C1' H. Two H atoms were then attached to the C1' methine to complete the N-methylated nucleobases (Figure 2) [5]. As mentioned in Section 2.1, the designation “A_1_T_1_”, “G_2_X_2_”, and “G_3_C_3_” were used for the consecutive base pairs (Figure 2 and Figure 3). 

Gaussian 03 (Gaussian Inc., Wallingford, CT, USA) [19] was used to calculate the destabilization energy of “A_1_T_1_” (*ΔE_1_*) of each G:X complex *in vacuo* at the B3LYP/6-31G** level. Moreover, to estimate the energy of each “A_1_T_1_” base pair in water, the Onsanger reaction field model and a dielectric constant of 78.39 were used to calculate the SCRF value of each of these base pairs. The destabilization energies of each “G_3_C_3_” (*ΔE_3_*) were calculated *in vacuo* and in water, just as *ΔE_1 _*values were calculated*.* The destabilization energies of “A_1_T_1_ + G_3_C_3_” (*ΔE_1 + 3_*) were calculated *in vacuo* and in water. The calculated destabilization energies (*ΔE_1_*,* ΔE_3_*, and *ΔE_1 + 3_*) are defined in Equations (1)–(3).

*ΔE_1_* = *E*(“A_1_T_1_” of G:X complex (X = C)) –*E*(“A_1_T_1_” of G:X complex (X = Oz, *S*-Ia, *R*-Ia, *S*-Sp or *R*-Sp)) (1)

*ΔE_3_* = *E*(“G_3_C_3_” of G:X complex (X = C)) –*E*(“G_3_C_3_” of G:X complex (X = Oz, *S*-Ia, *R*-Ia, *S*-Sp or *R*-Sp)) (2)

*ΔE_1 + 3_* = *E*(“A_1_T_1_+G_3_C_3_” of G:X complex (X = C)) –*E*(“A_1_T_1_+G_3_C_3_” of G:X complex (X = Oz, *S*-Ia, *R*-Ia, *S*-Sp or *R*-Sp)) (3)

### 3.3. Calculating the Degree of Distortion

**Figure 5:**
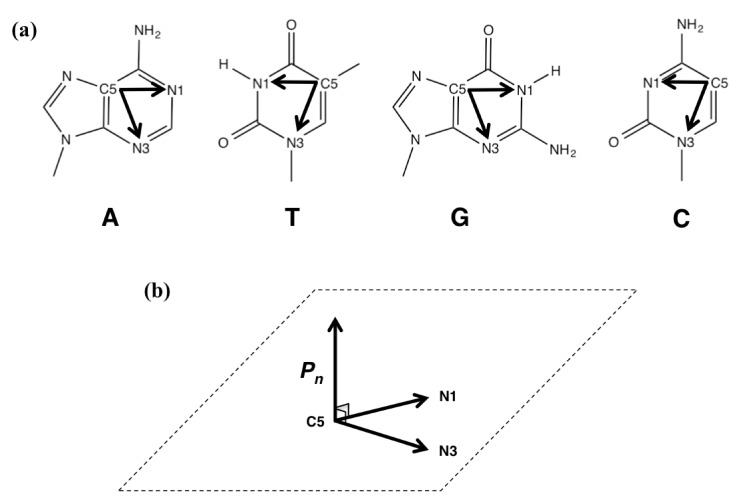
(**a**) Vector ***C5N1*** and vector ***C5N3*** in A, T, G, or C. (**b**) Normal vector ***P****_n_* was calculated from ***C5N1*** and vector ***C5N3*** (Equation (4)).

We calculated values for the vectors ***C5N1*** and ***C5N3*** using the C5 (x_C5_, y_C5_, z_C5_), N1 (x_N1_, y_N1_, z_N1_), N3 (x_N3_, y_N3_, z_N3_) atoms for each of five bases A_1_, T_1_, G_2_, G_3,_ and C_3_ (Figure 5a). The normal vector ***P****_n_* was calculated from vectors ***C5N1*** and ***C5N3*** (Equation (4)) (Figure 5b), and *n* represented A_1_, T_1_, G_2_, G_3,_ or C_3_:

***P****_n_* (x_n_, y_n_, z_n_) = ***C5N1*** × ***C5N3*** = (x_N1_– xC5, y_N1_ – y_C5_, z_N1_– z_C5_) × (x_N3_– x_C5_, y_N3_– y_C5_, z_N3_– z_C5_) = ((y_N1_– y_C5_)•(z_N3_– z_C5_)−(z_N1_– z_C5_)•(y_N3_– y_C5_), (z_N1_– z_C5_)•(x_N3_– x_C5_) – (x_N1_–x_C5_)•(z_N3_– z_C5_), (x_N1_– x_C5_)•(y_N3_– y_C5_) − (y_N1_– y_C5_)•(x_N3_– x_C5_)) (4)

Next, we calculated the dihedral angles between G_2_ and A_1_ (*θ* (G_2_–A_1_)), G_2_ and T_1_ (*θ* (G_2_–T_1_)), A_1_ and T_1_ (*θ* (A_1_–T_1_)), G_2_ and G_3_ (*θ* (G_2_–G_3_)), G_2 _and C_3_ (*θ* (G_2_–C_3_)), or G_3 _and C_3_ (*θ* (G_3_–C_3_)) (Figure 4) using the two respective normal vectors (Equations (5)–(10)):

*θ* (G_2_–A_1_) = arccos (***P****_G2_*• ***P****_A1_*/|***P****_G2_*| |***P****_A1_*|) (5)

*θ* (G_2_–T_1_) = arccos (***P****_G2_* • ***P****_T1_*/|***P****_G2_*| |***P****_T1_*|) (6)

*θ* (A_1_–T_1_) = arccos (***P****_A1_* • ***P****_T1_*/|***P****_A1_*| |***P****_T1_*|) (7)

*θ* (G_2_–G_3_) = arccos (***P****_G2_* • ***P****_G3_*/|***P****_G2_*| |***P****_G3_*|) (8)

*θ* (G_2_–C_3_) = arccos (***P****_G2_* • ***P****_C3_*/|***P****_G2_*| |***P****_C3_*|) (9)

*θ* (G_3_–C_3_) = arccos (***P****_G3_* • ***P****_C3_*/|***P****_G3_*| |***P****_C3_*|) (10)

As an index of the distortion of the DNA duplex, we defined the degree of distortion (*δ_1_* and *δ_3_*) (Equations (11) and (12)).

*δ*_1_ = *θ* (G_2_–A_1_) + *θ* (G_2_–T_1_) + *θ* (A_1_–T_1_) (11)

*δ*_3_ = *θ* (G_2_–G_3_) + *θ* (G_2_–C_3_) + *θ* (G_3_–C_3_) (12)

## 4. Conclusions 

Previous experimental results show that incorporation of guanine is more efficient when the template base is Oz than when it is Ia or Sp; however, the difference between Oz and Ia or Sp is not explained by the stabilities of G:Oz, G:Ia, and G:Sp as calculated *ab initio*. Thus, we focused on differences among G:Oz, G:Ia, and G:Sp with regard to planarity of base pair, and tried to explain the previous experimental results based on differences in distortion of DNA containing these DNA lesion.

Using models of 3-base-pair DNA duplexes that each contained a variable central “G_2_X_2_” base pair, we calculated the destabilization energies of the parts common to each model relative to energy of the structure derived from DNA containing a central G_2_C_2_ base pair. For the five X_2 _variants, we found that the stabilities of the “A_1_T_1_” base pairs on the 5'-side of X_2_ were ordered as follows: *R*-Ia > Oz > *S*-Ia > *S*-Sp > *R*-Sp, and that the stabilities of the “G_3_C_3_” base pairs on the 3'-side of X_2_ were ordered as follows: Oz > *S*-Ia > *S*-Sp > *R*-Ia > *R*-Sp. These data could not explain the previous experimental results. We also calculated the stability of the structure including both adjacent base pairs and excluding the central “G_2_X_2_” (“A_1_T_1_ + G_3_C_3_”); we found that, for the five X_2 _variants, the stability of “A_1_T_1_ + G_3_C_3_” was ordered as follows: Oz > *S*-Ia > *R*-Ia >* S*-Sp > *R*-Sp. The previous experimental results could be explained by considering the stability of the structure when accounting for both the 5' and 3' sides of X_2_. 

Additionally, we parameterized the distortion of DNA duplex in order to examine the effects of Oz, *S-*Ia, *R-*Ia *S-*Sp, or *R*-Sp. The degree of distortion at the 5'-side of X_2_ (*δ_1_*) or at the 3'-side of X_2_ (*δ_3_*) could not explain the stability of “A_1_T_1_” or “G_3_C_3_”, respectively, or the previous experimental results. However, compared with the structure of DNA containing a central G_2_C_2_ base pair, the total degree of distortion (“*δ_1_ +* *δ_3_*”) at the 5' and 3' sides of X_2_ were ordered as follows for the five X_2 _variants: *R*-Sp* >> S*-Sp >> *R*-Ia > *S*-Ia >> Oz. Values “*δ_1_ +* *δ_3_*” coincided with the stabilities of “A_1_T_1_ + G_3_C_3_”, and either calculation could account for the previous experimental results. Taken together, our discussions indicated that DNA containing a nonplanar lesion was susceptible to distortion and to destabilization, and consequently the bypass efficiency of DNA polymerase at the more nonplanar lesion was reduced relative to that at the more planar lesion. We showed that calculating energies of the structure accounting for both base pairs adjacent to G:Oz, G:Ia, or G:Sp was sufficient to evaluate the stability of the DNA duplex containing any of these base pairs. The distortion of the structure of DNA a duplex containing a central G:Oz was smaller than that of DNA containing a G:Ia or G:Sp; therefore, a G:Oz base pair was more easily bypassed by DNA polymerase than was a G:Ia or G:Sp base pair.

In this study, to minimize by MM calculation, we used the models contained not only the residues of protein and base pairs but also the sugar and phosphate components. There are not, however, the sugar and phosphate components in the DNA models used QM calculation of the stability, because models containing these components cannot calculate in a realistically acceptable amount of time due to many atoms. Therefore, to explain the more accurate effect of distortion caused these DNA damages, it will need to be considered how distortion within the bases can lead to changes in sugar conformation in the future.
